# A Review of 2010 for *PLoS Computational Biology*

**DOI:** 10.1371/journal.pcbi.1002003

**Published:** 2011-01-27

**Authors:** Rosemary Dickin, Cecy Marden, Andrew M. Collings, Ruth Nussinov, Philip E. Bourne

**Affiliations:** 1 Public Library of Science, Cambridge, United Kingdom; 2 National Cancer Institute, SAIC-Frederick, Maryland, United States of America; 3 Tel Aviv University, Tel Aviv, Israel; 4 Department of Pharmacology, University of California San Diego, La Jolla, California, United States of America; 5 Skaggs School of Pharmacy and Pharmaceutical Sciences, University of California San Diego, La Jolla, California, United States of America

*PLoS Computational Biology* celebrated its fifth anniversary in 2010, and all in our community, either as readers, authors, or editors, should take pride in what has been accomplished in such a short space of time. In the past year we received 1,403 new Research Articles, a 295% increase from our first year of operation in 2005–2006 and a 17% increase over 2009. Of the articles submitted in 2010, 875 (62%) were rejected, and 70% of these were before review. We have seen growth not only in submissions, but in readership as well. Currently, around 16,000 readers receive the electronic table of contents, a 14% increase over the previous year. We published 392 Research Articles this year, along with 23 “front section” articles (Reviews, Perspectives, Education), down from 33 in the previous year. Eighty Associate Editors handled the combined submissions, with a total of 26 new editors joining this past year and six departing. We are proud to say that virtually every editor we asked to join accepted, a testament to how our community values the journal. These editors worked with more than 180 guest editors and 1,800 reviewers to handle the submissions, and we are of course very grateful for their support (Table S1).

Table 1 provides a list of Research Articles we have published since 2005 through October 2010 that have accrued over 10,000 downloads and shows the diversity of highly accessed papers published by the journal. Note that these are downloads from the PLoS Web site only, and do not include downloads from PubMed Central. Readers are free to review download statistics for all research and non-research articles published across the PLoS journals through the Microsoft Excel spreadsheet that can be found at http://www.ploscompbiol.org/static/plos-alm.zip. Individual article metrics and comments are available from the respective tabs associated with each article.

**Table 1:** List of published Research Articles that have accrued over 10,000 downloads since launch.

Total Views/Downloads	Citation	URL
21,014	Yang S, Roux B (2008) Src Kinase Conformational Activation: Thermodynamics, Pathways, and Mechanisms. PLoS Comput Biol 4(3): e1000047. doi:10.1371/journal.pcbi.1000047	http://www.ploscompbiol.org/doi/pcbi.1000047
20,177	Wen Q, Chklovskii DB (2005) Segregation of the Brain into Gray and White Matter: A Design Minimizing Conduction Delays. PLoS Comput Biol 1(7): e78. doi:10.1371/journal.pcbi.0010078	http://www.ploscompbiol.org/doi/pcbi.0010078
18,313	Rapoport BI (2010) Metabolic Factors Limiting Performance in Marathon Runners. PLoS Comput Biol 6(10): e1000960. doi:10.1371/journal.pcbi.1000960	http://www.ploscompbiol.org/doi/pcbi.1000960
18,152	Grün D, Wang Y-L, Langenberger D, Gunsalus KC, Rajewsky N (2005) microRNA Target Predictions across Seven Drosophila Species and Comparison to Mammalian Targets. PLoS Comput Biol 1(1): e13. doi:10.1371/journal.pcbi.0010013	http://www.ploscompbiol.org/doi/pcbi.0010013
17,525	Allesina S, Pascual M (2009) Googling Food Webs: Can an Eigenvector Measure Species' Importance for Coextinctions? PLoS Comput Biol 5(9): e1000494. doi:10.1371/journal.pcbi.1000494	http://www.ploscompbiol.org/doi/pcbi.1000494
17,425	George D, Hawkins J (2009) Towards a Mathematical Theory of Cortical Micro-circuits. PLoS Comput Biol 5(10): e1000532. doi:10.1371/journal.pcbi.1000532	http://www.ploscompbiol.org/doi/pcbi.1000532
16,482	Li C-Y, Mao X, Wei L (2008) Genes and (Common) Pathways Underlying Drug Addiction. PLoS Comput Biol 4(1): e2. doi:10.1371/journal.pcbi.0040002	http://www.ploscompbiol.org/doi/pcbi.0040002
14,398	Brinkworth RSA, O'Carroll DC (2009) Robust Models for Optic Flow Coding in Natural Scenes Inspired by Insect Biology. PLoS Comput Biol 5(11): e1000555. doi:10.1371/journal.pcbi.1000555	http://www.ploscompbiol.org/doi/pcbi.1000555
13,774	Pedersen JS, Bejerano G, Siepel A, Rosenbloom K, Lindblad-Toh K, et al. (2006) Identification and Classification of Conserved RNA Secondary Structures in the Human Genome. PLoS Comput Biol 2(4): e33. doi:10.1371/journal.pcbi.0020033	http://www.ploscompbiol.org/doi/pcbi.0020033
12,884	Kitzbichler MG, Smith ML, Christensen SR, Bullmore E (2009) Broadband Criticality of Human Brain Network Synchronization. PLoS Comput Biol 5(3): e1000314. doi:10.1371/journal.pcbi.1000314	http://www.ploscompbiol.org/doi/pcbi.1000314
12,635	Scheeff ED, Bourne PE (2005) Structural Evolution of the Protein Kinase–Like Superfamily. PLoS Comput Biol 1(5): e49. doi:10.1371/journal.pcbi.0010049	http://www.ploscompbiol.org/doi/pcbi.0010049
12,527	Engelhardt BE, Jordan MI, Muratore KE, Brenner SE (2005) Protein Molecular Function Prediction by Bayesian Phylogenomics. PLoS Comput Biol 1(5): e45. doi:10.1371/journal.pcbi.0010045	http://www.ploscompbiol.org/doi/pcbi.0010045
12,015	Manoonpong P, Geng T, Kulvicius T, Porr B, Wörgötter F (2007) Adaptive, Fast Walking in a Biped Robot under Neuronal Control and Learning. PLoS Comput Biol 3(7): e134. doi:10.1371/journal.pcbi.0030134	http://www.ploscompbiol.org/doi/pcbi.0030134
11,359	Barker D, Pagel M (2005) Predicting Functional Gene Links from Phylogenetic-Statistical Analyses of Whole Genomes. PLoS Comput Biol 1(1): e3. doi:10.1371/journal.pcbi.0010003	http://www.ploscompbiol.org/doi/pcbi.0010003
11,160	Itan Y, Powell A, Beaumont MA, Burger J, Thomas MG (2009) The Origins of Lactase Persistence in Europe. PLoS Comput Biol 5(8): e1000491. doi:10.1371/journal.pcbi.1000491	http://www.ploscompbiol.org/doi/pcbi.1000491
10,645	Pinto N, Cox DD, DiCarlo JJ (2008) Why is Real-World Visual Object Recognition Hard? PLoS Comput Biol 4(1): e27. doi:10.1371/journal.pcbi.0040027	http://www.ploscompbiol.org/doi/pcbi.0040027
10,477	Pinto N, Doukhan D, DiCarlo JJ, Cox DD (2009) A High-Throughput Screening Approach to Discovering Good Forms of Biologically Inspired Visual Representation. PLoS Comput Biol 5(11): e1000579. doi:10.1371/journal.pcbi.1000579	http://www.ploscompbiol.org/doi/pcbi.1000579
10,447	Beard DA (2005) A Biophysical Model of the Mitochondrial Respiratory System and Oxidative Phosphorylation. PLoS Comput Biol 1(4): e36. doi:10.1371/journal.pcbi.0010036	http://www.ploscompbiol.org/doi/pcbi.0010036
10,342	Siddharthan R, Siggia ED, van Nimwegen E (2005) PhyloGibbs: A Gibbs Sampling Motif Finder That Incorporates Phylogeny. PLoS Comput Biol 1(7): e67. doi:10.1371/journal.pcbi.0010067	http://www.ploscompbiol.org/doi/pcbi.0010067
10,244	Chickarmane V, Troein C, Nuber UA, Sauro HM, Peterson C (2006) Transcriptional Dynamics of the Embryonic Stem Cell Switch. PLoS Comput Biol 2(9): e123. doi:10.1371/journal.pcbi.0020123	http://www.ploscompbiol.org/doi/pcbi.0020123
10,075	Zhou X, Ruan J, Wang G, Zhang W (2007) Characterization and Identification of MicroRNA Core Promoters in Four Model Species. PLoS Comput Biol 3(3): e37. doi:10.1371/journal.pcbi.0030037	http://www.ploscompbiol.org/doi/pcbi.0030037

Data correct as of October 13, 2010.

In 2010, we launched two new features to enrich the journal: “The Roots of Bioinformatics” and “PLoS Conference Postcards”. The Roots of Bioinformatics was eloquently introduced by the Series Editor, David B. Searls, in June [1] and was followed in July by Russell F. Doolittle’s insightful reflections on the roots of protein evolution, which went back as far as the 1950s when chemistry, rather than computers, ruled [2]. More such reflections will follow in 2011. Conference Postcards act as a counterpoint to the rich roots retrospectives by providing current views of the field of computational biology, as young scientists present crisp perspectives on what they perceive as conference highlights. We published Postcards from January’s Pacific Symposium on Biocomputing (PSB) meeting held in Hawaii [3] and from the Intelligent Systems for Molecular Biology (ISMB) meeting held in Boston in July [4]. At the latter we learnt about various sessions held at ISMB, namely the Highlights session, the ISCB Student Council Symposium’s “speed dating” event, and reports from Satellite meetings. We look forward to digging deeper and receiving Postcards from further afield in 2011.

*PLoS Computational Biology* continues its strong relationship with the International Society for Computational Biology (ISCB) through postings on its Web site and activities at ISMB. At ISMB 2010 in Boston, *PLoS Computational Biology* ran a Workshop entitled “Where and How to Get Published” in which we endeavored to make the path to getting published a little less inscrutable. The first half was led by journal co-founders Philip E. Bourne and Steven E. Brenner and provided guidelines on how to write a good paper, and the second half included questions and advice from editors and authors from a range of career stages, which resulted in a broad discussion of what journals want and the state of publishing today. Presenters’ materials from the Workshop are available on the new PLoS Blog (http://blogs.plos.org/plos/2010/10/materials-from-plos%E2%80%99-workshop-at-ismb-2010/).

We have three major goals for 2011. First, to reduce the time to decision for submitted manuscripts, which currently averages 10 days for those papers rejected before review and 40**–**50 days for those reviewed. Second, to introduce a new section called “Editors’ Outlook”, which are invited mini-reviews from members of our Editorial Board who will provide insights into their respective fields, discussing what is hot and what we can expect going forward. Collectively, these will provide an ongoing and insightful look into the broad and rapidly expanding field of computational biology—a field of endeavor the journal is proud to serve.

Specifically, current experimental techniques are leading to an unprecedented increase in the rate at which data are becoming available. When combined with the vast growth in computational power, we can expect rapid growth in computational papers. Computational biology is the area that helps in organizing the data, in making sense of observations, and in using these to make experimentally testable predictions. Our third goal is to keep abreast of these developments and keep *PLoS Computational Biology* the number one journal in the field.

## Supporting Information

Table S1 Guest Editors and reviewers for *PLoS Computational Biology* in 2010.
(XLS)
